# Characterising youth with callous–unemotional traits and concurrent anxiety: evidence for a high-risk clinical group

**DOI:** 10.1007/s00787-017-1086-8

**Published:** 2017-12-08

**Authors:** Charlotte A. M. Cecil, Eamon J. McCrory, Edward D. Barker, Jo Guiney, Essi Viding

**Affiliations:** 1 Department of Psychology, Institute of Psychiatry, King’s College London, London, UK; 2 Division of Psychology and Language Sciences, University College London, 26 Bedford Way, London, WC1H 0AP UK; 3 Department of Psychology, Royal Holloway, University of London, Egham, UK

**Keywords:** Callous–unemotional traits, Anxiety, Childhood maltreatment, Psychopathology, Adolescence

## Abstract

Growing evidence supports the existence of two variants of youth with high callous–unemotional (CU) traits who present with markedly different risk profiles and outcomes, with potential implications for risk assessment and treatment formulation. So far, studies have identified variants of CU youth mainly using data-driven cluster approaches based on levels of CU traits and co-occurring anxiety. Yet, the extent to which this knowledge may be translated into clinical practice is unclear. To this end, the present study employed a severity-based, cut-off approach to systematically characterise CU groups across a range of clinically informative domains, including trauma history, psychiatric symptomatology, affective functioning, attachment style and behavioural risk. Analyses were based on multi-rated data from a community sample of high-risk youths (*n* = 155, *M* = 18 years). Consistent with previous studies, we found that, whereas variants show comparable levels of antisocial behaviour, those who present with both high CU and high anxiety report more severe childhood maltreatment, psychological distress, ADHD symptomatology and behavioural risk—including substance use, suicidal ideation and unsafe sex. In addition, these youth show greater attachment insecurity and affective dysregulation, as indexed by levels of irritability and alexithymia. Together, findings indicate that (1) trauma history is a key factor that differentiates variants of CU youth high vs. low on anxiety, and (2) differences in individual functioning across variants point to the need for tailored clinical assessment tools and intervention strategies. Importantly, the present findings indicate that variants of CU youth can be meaningfully differentiated using cut-off based approaches that parallel methods used in clinical assessments.

## Introduction

In the DSM-5, callous–unemotional (CU) traits—referred to as ‘Limited Prosocial Emotions’—feature as a new diagnostic specifier for conduct disorder, to enable the identification of a particularly severe subgroup of youth at increased risk for early-onset and persistent antisocial behaviour [1]. CU traits are defined by a core set of affective features (paralleling the affective dimension of adult psychopathy), which include low capacity for empathy, lack of guilt and remorse, callousness and shallow affect [2]. Compared to other antisocial youth, those with high CU traits show marked differences in neurocognitive, emotional and behavioural functioning, including difficulties in social-information processing [3], under-arousal to empathy-inducing stimuli [4], disruptions in affective theory of mind [5], lower sensitivity to punishment [1] as well as alterations in brain regions involved in emotion and learning (e.g. amygdala, PFC; [2]). Together, these features are thought to contribute to the more violent, chronic and recidivistic pattern of antisocial behaviour displayed by youth with high CU traits, and represent an important target for intervention.

It is also becoming increasingly clear that not all youth with high CU traits are the same. Rather, they can present with different levels of co-occurring anxiety [6, 7]. This is akin to what has been observed in adults with psychopathy [8] and is thought to reflect the existence of two variants with potentially distinct aetiologies—a theory first put forward by Karpman [9] and Cleckley [10] in 1941. Specifically, CU traits accompanied by *low* levels of anxiety (*CU*−*Anx* variant) are thought to be associated with substantial developmental genetic risk, whereas CU traits accompanied by *high* levels of anxiety (*CU*+*Anx* variant) are thought to be associated primarily with environmental trauma [11, 12]. The two variants are indistinguishable based on CU traits alone (i.e. they can be thought of as ‘behavioural phenocopies’), but the *CU*+*Anx* variant is associated with more severe pre- [7, 13] and post-natal [14, 15] adversity, with the most consistent evidence relating to childhood maltreatment [16–20]. Furthermore, variants have been shown to differ markedly in presentation across a range of domains, including comorbid psychiatric symptomatology [7, 14, 15, 21, 22], impulsivity [19], self-control [23], empathy [24], personality traits [25], expression of aggression [26], negative affect [20], emotional lability [27], emotional processing [6, 17, 28, 29], behavioural risk [15, 18] and biological function [13, 26, 30].

Given that CU levels are currently used to inform risk assessment and treatment options with antisocial youth [1], the existence of variants may carry important implications for clinical practice [17]. So far, studies have primarily identified variants of CU youth using state-of-the-art clustering approaches, which are hypothesis-free and person-centred [6, 7, 14–18, 26–28, 30–32]. Together, these reports have been invaluable in demonstrating that individuals naturally cluster into groups based on their on levels of CU and anxiety—providing strong, data-driven evidence validating the existence of two variants of CU youth. However, clustering approaches are not practical in clinical settings, where treatment and risk assessment decisions are typically based on variable-centred, severity-based thresholds. A handful of other studies have examined these traits continuously, as opposed to comparing groups, in order to establish whether presence of anxiety or trauma history moderates the association between CU traits and outcomes, such as empathy [24] or emotional recognition [33]. While such an approach has the advantage of modelling the full range of scores, lending useful insights into the dimensional relationship between CU and anxiety, it is particularly difficult to implement in a clinical setting.

As an alternative, a small set of studies have shown that simpler cut-off approaches (e.g. based on average scores) can be successfully employed to compare variants on specific outcomes, yielding results that are consistent with those derived from cluster-based approaches. For example, in a Romanian sample of incarcerated males (*n* = 125, age 14–18 years), Rosan and colleagues [19] used the sample average score of CU traits and anxiety as a cut-off threshold to classify youth as either CU+Anx, CU−Anx or a control group low on both dimensions. The authors found that the CU+Anx group showed significantly higher levels of impulsivity and emotional dysregulation (e.g. anger, suicidal ideation, thought disturbance) compared to both the CU−Anx and control group. In another study based on male juvenile offenders (*n* = 238, age 14–19 years), Sharf and colleagues [20] used a median-split approach to create the same three groups (i.e. CU+Anx, CU−Anx, control group) and found that the CU+Anx group reported greater exposure to negative life events (especially violence exposure in the home and community) as well more severe post-traumatic symptoms compared to the other two groups. While promising, these studies have focussed exclusively on male youth offenders, so that more work is needed to test whether severity-based approaches can meaningfully differentiate variants in non-forensic, multi-gender populations across a wider range of clinically informative domains.

A further question with important clinical implications is how *CU*+*Anx* youth compare not only to their *CU*−*Anx* counterparts, but also to youth who present with high anxiety alone (*Anxious* group). Contrasting these two groups is necessary in order to clarify whether (1) *CU*+*Anx* youth experience a ‘double hit’ of negative outcomes associated with two relatively independent dimensions of psychopathology; or (2) whether the combination of high CU and Anxiety indexes a particularly high-risk group of youth who show *additional* vulnerabilities compared to those who present with either CU or Anxiety alone. Because studies to date (both cluster-based and severity-based) have generally contrasted variants of CU youth to a single, generic comparison group (i.e. not disaggregated by level of anxiety), it has not been possible to systematically address this question. To our knowledge, only one study based on a community sample of adolescents has compared the *CU*+*Anx* group to a reference group who show comparable levels of anxiety [31]. Interestingly, the authors reported that although the *Anxious* group displayed lower levels of CU traits and antisocial behaviour compared to the *CU*+*Anx* variant, the groups presented similarly in other domains, such as low self-esteem. Furthermore, the *Anxious* group consisted primarily of girls, which may explain the failure to identify this subgroup in prior studies that have typically focused on juvenile male offender samples. The study, however, did not compare groups on trauma history, psychiatric risk, and affective functioning—key clinical domains that need systematic investigation if we are to more fully understand the nature of the *CU*+*Anx* variant.

## The present study

The aim of the present study was to comprehensively characterise variants of CU traits in a community sample of high-risk youth. Specifically, we investigated whether variants of CU youth (i.e. *CU*−*Anx* vs *CU*+*Anx*) identified using a variable-centred, median-based approach differ across: (1) previously validated domains, including childhood maltreatment history, (multi-rated) psychiatric symptoms, and behavioural risk markers; as well as (2) novel functional domains, including attachment style and affective functioning (irritability and alexithymia). To improve the specificity of any conclusions about these groups we also compared both CU variant groups with two clinically relevant comparison groups a *Low* group (low on both CU and anxiety) and an *Anxious* group (low on CU but high in anxiety). Based on previous studies using cluster-based analyses as well as those that have used severity-based cut-offs, we predicted that, relative to youth only high in CU traits (*CU*−*Anx*), those with high CU and high anxiety (*CU*+*Anx*) would be characterised by: (1) more severe experiences of childhood maltreatment; (2) greater levels of psychological distress and psychiatric symptomatology; (3) significantly elevated behavioural risk markers; but (4) similar levels of externalising problems. Given the lack of prior research, no a priori hypotheses were made regarding associations with attachment style or affective functioning (as indexed by levels of irritability and alexithymia) between variants of CU youth. Compared to the *Anxious* group, we expected that *CU*+*Anx* youth would show higher levels of externalising problems (in line with previous studies [31]); however, no specific predictions were made for maltreatment history, psychiatric risk and affective functioning, as these domains have not been previously examined with *Anxious* vs *CU*+*Anx* groups.

## Method

### Participants

The current sample draws from a larger study (*n* = 204) examining the effects of developmental adversity on individual functioning amongst socially deprived youth aged 16–24 years (mean age 18 years). Of note, we refer to our sample as ‘youth’, as it is (1) in line with the term used by international organisations (e.g. UN) to describe individuals aged 15–24; and (2) consistent with the extant literature on variants of CU youth, which is primarily focused on youth populations (e.g. [17–20, 24, 25]). Only participants for whom information was available for both CU traits and anxiety were included in the present study (*n* = 155). These youth were recruited via multiple channels in order to capture varying exposure to adversity, including inner-city colleges (*n* = 71, 46%) and a charity providing services and support to vulnerable, self-referred youth (*n* = 84, 54%). Of the total sample, 80% of participants were under the age of 20 years (*M* = 18) and 54% were females (*N* = 84). The sample was ethnically diverse, with 52% Caucasian, 42% Black, 6% ‘Other’ participants.

### Procedure

All procedures performed were in accordance with the ethical standards of the UCL Research Ethics Committee (ID No. 2462/001) and with the 1964 Helsinki declaration and its later amendments or comparable ethical standards. Youth from the charity were introduced to the research by a member of staff, and, if interested, were provided information about the study by one of the research team on site. As a result, all youth who met with the researchers had shown interest in the study and agreed to participate. After the testing session, each participant’s key worker completed a questionnaire booklet. A key worker is a member of staff of the charity who is assigned to each client upon referral in order to assist in the delivery of services as well as to provide socio-emotional and practical support. In schools, youth initially received information during a brief presentation at a school assembly. Information sheets and consent forms were then distributed to students who had attended the presentation. Those students who were interested in taking part completed the consent form and returned it to the researchers. As a result, researchers met exclusively with students who were interested in participating and had provided informed consent stating that they were willing to take part in the study. After the consent forms were returned, a timetable was circulated by the Deputy Head of the schools to teachers in the participants’ class year, in order to (1) select slots that would be the least disruptive to each participant’s class schedule; and (2) identify which teachers knew each participant best and thus could be asked to fill in the questionnaire booklet after the testing session had taken place. Out of the participants who initially consented to take part in the study, 89.6% attended the agreed time slots and completed the testing session. After the testing session, the teachers most familiar with each participant completed the questionnaire booklet. Of note, 88% of informants (i.e. key workers/teachers) reported knowing the participant well (i.e. ‘a little’ = 12%; ‘moderately well’ = 54%; ‘very well’ = 34%). Informed consent was obtained from all participants included in the study. Testing took place in a quiet room within the charity or the young person’s school depending on recruitment source. Participants from the charity were compensated for their time individually; however, students recruited from schools received group compensation for school equipment or a final year party in line with head-teacher preferences. Additional details of the recruitment procedures are available elsewhere [34].

### Measures

#### Socio-demographic characteristics

Data on age, sex, ethnicity and IQ were collected from all participants. Cognitive ability was assessed using the two-subtest version of the Wechsler Abbreviated Scale of Intelligence (WASI; [35]), with all participants scoring within the 70–125 range. Participant postcode information was used to obtain a census-derived and area-weighted Index of Multiple Deprivation (IMD; [36]) score, an aggregate measure of neighbourhood deprivation. Higher values indicate older age, female gender, non-white ethnicity, higher cognitive ability and greater neighbourhood deprivation.

#### Indicator variables

##### Callous–unemotional traits

CU traits were measured using the well-validated Inventory of Callous Unemotional traits (ICU; [37]), based on informant ratings (i.e. teachers or key workers, depending on recruitment site). The ICU contains 24 items rated on a 4-point scale from ‘*not at all true*’ to ‘*definitely true*’. The items cluster into three subscales, which show adequate internal reliability in our sample: callous (*α* = 0.79), uncaring (*α* = 0.88), and unemotional (*α* = 0.73). The total ICU score was used to identify CU groups (*α* = 0.79).

##### Anxiety

Participants completed the anxiety subscale of the Trauma Symptom Checklist for Children (TSCC-A; [38]). The TSCC-A is a 44-item self-report inventory that includes 5 clinical scales (anxiety, depression, post-traumatic stress, anger and dissociation) and 2 validity scales (under- and hyper-response). Each item is rated on a 4-point scale from ‘*never*’ to ‘*almost all of the time*’. Of note, although the TSCC-A is designed to measure common sequelae of trauma exposure, the anxiety scale makes no reference to traumatic events. Rather, items tap into unspecific symptoms of general anxiety, such as “feeling afraid something bad may happen”, “worrying about things” and “feeling nervous or jumpy inside” (9 items; *α* = 0.86).

#### Maltreatment history

Participants completed the Childhood Trauma Questionnaire (CTQ; [39]), a widely used 28-item self-report measure screening for experiences of maltreatment “while growing up”. Items are rated on a 5-point scale from ‘*never true*’ to ‘*very often true*’ (e.g. “people in my family hit me so hard that it left me with bruises or marks”). The CTQ comprises five subscales measuring emotional abuse, physical abuse, sexual abuse, emotional neglect and physical neglect. The scales show acceptable internal consistency in our sample (*α* = 0.70–0.97). Higher scores represent more severe experience of childhood maltreatment.

#### Markers of individual functioning

##### Psychiatric symptoms

Psychiatric symptomatology was assessed using both self- and informant-report measures. Symptoms of depression, anger, post-traumatic stress and dissociation were assessed using the self-report clinical scales from the TSCC-A, as described above (*α* = 0.84–0.87). In addition, informants completed six subscales from the DSM-IV-based Adolescent Symptom Inventory (ASI-4; [40]) to assess symptoms of emotional and behavioural disorders, including generalised anxiety disorder (GAD), major depressive disorder (MDD), oppositional defiant disorder (ODD), conduct disorder (CD), antisocial personality disorder (ASPD) and attention-deficit hyperactivity disorder (ADHD). Each scale contained between 7 and 9 items (*α* = 0.89–0.94). Each item is rated on a 3-point scale from ‘*not true*’ to ‘*certainly true*’.

##### Behavioural risk

Multiple domains of behavioural risk-taking were assessed based on self-reported measures. Substance use was assessed via the Alcohol Use Disorders Identification Test (AUDIT; [41]) and the Drug Use Disorders Identification Test (DUDIT; [42]). The AUDIT and DUDIT include 10 and 11 items, respectively, measuring substance use, harmful use and symptoms of dependence. The first items are rated on a 5-point scale ranging from ‘*never*’ to ‘*daily or almost daily*’. The last two items from each scale are rated on a 3-point scale and are coded as 0 (‘*no*’), 2 (‘*yes, but not during the last year*’) or 4 (‘*yes, during the last year*’). Cronbach’s alphas for the AUDIT and DUDIT were 0.82 and 0.90, respectively. Participants were additionally administered three yes/no items from the Youth Risk Behaviour Survey (YRBS; [43]). The first two items asked about suicidal ideation (“During the past 12 months, did you ever seriously consider attempting suicide”) and attempted suicide (“During the past 12 months, how many times did you actually attempt suicide?”; originally rated on a 5-point scale from ‘*0 times*’ to ‘*6 or more times*’ but collapsed due to low frequency of youth reporting multiple suicide attempts). The third item asked about sexual safety (“The last time you had sexual intercourse, did you or your partner use a condom or other contraceptive?”). Participants who reported not having had sexual intercourse were excluded from analysis of this item (*n* = 42).

##### Attachment style

The Experiences in Close Relationships Inventory (ECR; [44]) was used as a self-report measure of attachment. The ECR comprises of two 18-item scales, Anxiety (e.g. “I worry about being abandoned”; *α* = 0.92) and Avoidance (e.g. “I try to avoid getting to close to others”; *α* = 0.91). Here, we analysed categorical scores of attachment style derived using a median-based approach, consistent with Bartholomew and Horowitz’s model [37]. Participants were defined as (1) Secure, if scoring below midpoint on both scales (30% of sample); (2) Anxious, if above midpoint on the Anxiety scale only (16%); (3) Avoidant, if scoring above midpoint on the Avoidant scale only (26%), and (4) Disorganised, if scoring above midpoint on both (28%).

##### Affective functioning

Affective functioning was measured via self-report ratings of irritability and alexithymia. The Affective Reactivity Index (ARI; [45]) includes six items rated on a 3-point scale (‘*not true*’ to ‘*certainly true*’) and measures irritability over the past 6 months, including statements such as “easily annoyed by others” and “often lose temper”. Items were summed to form a total score, with adequate internal consistency (*α* = 0.88). The fist factor from the Toronto Alexithymia Scale (TAS-F1; [46]) was used to assess difficulty in the ability to identify one’s own feelings and to distinguish them from bodily sensations signalling emotional arousal. The scale comprises 7 items rated on a 5-point scale from ‘*I strongly disagree*’ to ‘*I strongly agree*’ (e.g. “when I am upset, I don’t know if I am sad, frightened, or angry”; *α* = 0.89).

### Statistical analysis

#### Step 1: defining groups

**Fig. 1:**
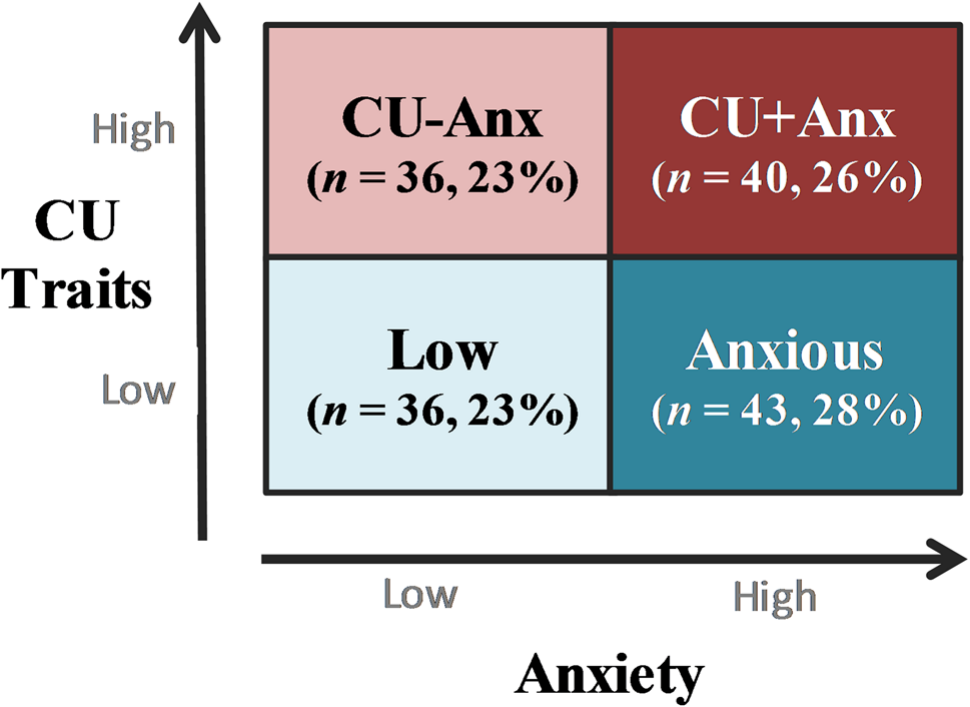
Study groups, including two variants of CU youth and two comparison groups

We disaggregated CU groups using a median-split approach, which resulted in four categorical groups (see Fig. 1): (1) ‘*Low*’, if scoring below midpoint on both measures of CU and anxiety (23%, *n* = 36); (2) ‘*Anxious*’, if scoring above midpoint on anxiety only (28%, *n* = 43); (3) ‘*CU*−*Anx*’, if scoring above midpoint on CU only (23%, *n* = 36); and (4) ‘*CU*+*Anx*’ if scoring above midpoint on both measures of CU and Anxiety (26%, *n* = 40). This approach parallels methods used in clinical assessments, which often rely on concrete cut-offs rather than categories achieved by data-driven approaches (e.g. cluster analyses). In line with previous findings (e.g. [24]), CU and anxiety measures did not correlate significantly when examined globally (*r* = 0.03). Of note, average levels of CU across the sample (*M* = 23.21; median = 22, see Table 1) were comparable to those observed in previous studies that have used the ICU to cluster variants of CU youth in mixed-gender samples, including community (e.g. *M* = 23.65; [25]) and juvenile offender populations (e.g. *M* = 22.33; [23]). Compared to these studies (both of which employed self-reports), CU levels across the variants identified here were slightly lower (i.e. *M*_CU−Anx_ = 31.09 compared to 32.30 in [25] and 33.24 in [23]; *M*_CU+Anx_ = 31.30 compared to 33.62 in [25] and 36.01 in [23]). Of note, the median-split approach used here makes it possible to compare the *CU*+*Anx* group to (1) a *CU*−*Anx* group, who shows comparable levels of CU levels but significantly lower levels of anxiety; and (2) an *Anxious* group, who instead shows comparable levels of anxiety but significantly lower levels of CU. Therefore, the method enables one to characterise similarities and differences between youth who present with *both* high CU and anxiety vs those who present with either one alone.

**Table 1:** Group comparisons on socio-demographic variables and maltreatment history

	Overall sample (*n* = 155)	Low CU		High CU		Omnibus test	Pair-wise contrasts	
		*Low* (*n* = 36)	*Anxious* (*n* = 43)	*CU*−*Anx* (*n* = 36)	*CU*+*Anx* (*n* = 40)		*CU*+*Anx* vs*. CU*−*Anx*	*CU*+*Anx* vs. *Anxious*
							Effect size [95% CI]	Effect size [95% CI]
*Socio-demographics*								
Sex (% female)	54.2	52.8	74.4	30.6	55.0	*X* ^2^ (3,155) = 15.23, *p* < 0.01^a^	^†^ OR = 2.84 [1.13, 7.14]	–
Ethnicity	80:65:10	27:8:1	22:17:4	17:17:2	14:23:2	*X* ^2^ (9,155) = 16.43, ns	–	–
Age, *M* (SD)	18.48 (2.11)	18.03 (2.16)	18.81 (2.16)	18.67 (2.03)	18.38 (2.07)	*F* (3,155) = 1.04, ns	–	–
IMD, *M* (SD)	28.22 (10.99)	25.01 (10.10)	29.21 (12.54)	29.31 (10.55)	29.20 (10.28)	*F* (3,155) = 1.36, ns	–	–
IQ, *M* (SD)	99.47 (10.95)	100.66 (9.70)	99.02 (12.48)	101.06 (9.14)	97.42 (11.75)	*F* (3,155) = 0.84, ns	–	–
*Indicator variables*								
CU traits (total), *M* (SD)	23.21 (9.53)	14.83 (4.35)	16.13 (4.38)	31.09 (6.03)	31.30 (6.68)	–	–	–
Callousness	4.76 (3.71)	2.42 (1.63)	2.30 (1.32)	7.33 (3.55)	7.17 (3.88)	–	–	–
Uncaring	11.78 (5.35)	6.97 (3.40)	8.56 (4.29)	15.88 (3.07)	15.96 (2.96)	–	–	–
Unemotional	6.72 (2.67)	5.50 (1.75)	5.28 (2.40)	8.00 (2.34)	8.25 (2.59)	–	–	–
Anxiety, *M* (SD)	6.74 (5.24)	2.42 (1.48)	10.16 (4.32)	2.72 (1.47)	10.55 (4.94)	–	–	–
*Maltreatment history*								
Emotional abuse, *M* (SD)	9.90 (5.08)	7.50 (2.83)	11.04 (5.23)	8.11 (4.31)	12.45 (5.68)	*X* ^2^(3,155) = 24.69, *p* < 0.001^a,b^	*** OR = 2.43 [1.47, 4.02]	–
Physical abuse, *M* (SD)	8.03 (4.88)	6.08 (1.64)	8.42 (5.28)	6.83 (3.41)	10.45 (6.32)	*X* ^2^(3,155) = 37.61, *p* < 0.001^b^	*** OR = 3.09 [1.81, 5.30]	–
Sexual abuse, *M* (SD)	5.97 (3.29)	5.22 (0.90)	6.55 (4.33)	5.47 (1.83)	6.47 (4.16)	*X* ^2^ (3,155) = 27.29, *p* < 0.001^a,b^	** OR = 2.82 [1.38, 5.76]	–
Emotional neglect, *M* (SD)	10.50 (4.76)	8.80 (3.54)	11.67 (5.07)	9.17 (4.18)	11.95 (5.19)	*X* ^2^ (3,155) = 9.44^†^	^†^ OR = 1.68 [1.02, 2.75]	–
Physical neglect, *M* (SD)	7.39 (3.45)	6.39 (2.60)	7.88 (3.85)	6.23 (2.34)	8.75 (3.98)	*X* ^2^ (3,155) = 22.05, *p* < 0.001^a,b^	*** OR = 3.01 [1.71, 5.29]	–
Total maltreatment, *M* (SD)	41.79 (17.23)	34.00 (8.99)	45.58 (18.93)	35.86 (12.65)	50.07 (19.87)	*X* ^2^ (3,155) = 25.86, *p* < 0.001^a,b^	*** OR = 2.33 [1.46, 3.08]	–

N.B. Analyses control for sex. Ethnicity = White:Black:Other. Omnibus test and pair-wise contrast are not performed for group-dependent variables (i.e. CU and Anxiety). Maltreatment history analysed using negative binomial regression. CU vs. Low do not differ in level of maltreatment. For the sake of clarity, tables presented only provide in-depth statistics for the contrasts of greatest interest (‘*CU*+*Anx*’ vs. ‘*CU*−*Anx*’ and ‘*CU*+*Anx*’ vs. ‘*Anxious*’). More detailed information about the other contrasts is available upon request

*OR* odds ratio, *IMD* Index of Multiple Deprivation, *CU* callous–unemotional

^†^ *p* < 0.05, ** *p* < 0.01, *** *p* < 0.001

^a^ *CU*−*Anx* vs *Anxious* contrast significant at *p* < 0.01

^b^ *CU*+*Anx* vs *Low* contrast significant at *p* < 0.01

#### Step 2: group comparisons

Group comparisons were performed using regression models, which differed depending on data distribution. Overdispersed count variables (maltreatment scores and substance use variables) were analysed using negative binomial regressions. Chi-square and logistic regressions were used for categorical data (sex, ethnicity, attachment style, suicidal ideation and attempt, unsafe sex). Linear regressions were used for all other variables (age, IMD, IQ, TSCC-A, ASI and affective functioning). For each analysis, we first report main effect statistics from the Omnibus test (i.e. *X* ^2^ statistic for negative binomial regressions and categorical data; *F* statistic for linear regressions). Pair-wise comparisons are then reported for all significant main effects, including effect sizes for significant pair-wise contrasts (odds ratio for negative binomial regressions and categorical data; Hedge’s *g* for linear regressions). To correct for inflated alphas resulting from multiple comparisons we set the alpha threshold at *p* <0.01. Analyses were performed on SPSS package v. 21 [47].

## Results

Descriptive statistics for socio-demographic variables are presented in Table 1. Groups did not differ across age, ethnicity, IQ and IMD. The ratio of males to females significantly differed across groups, *X* ^2^(3,155) = 15.23, *p* < 0.01. Over half of youth in the *CU*+*Anx* group were females compared to one third in the *CU*−*Anx* group. The number of females also differed markedly between *CU*−*Anx* and *Anxious* youth (30.6 vs. 74.4% females). As a result, all analyses included sex as a covariate.

### Maltreatment history

**Fig. 2:**
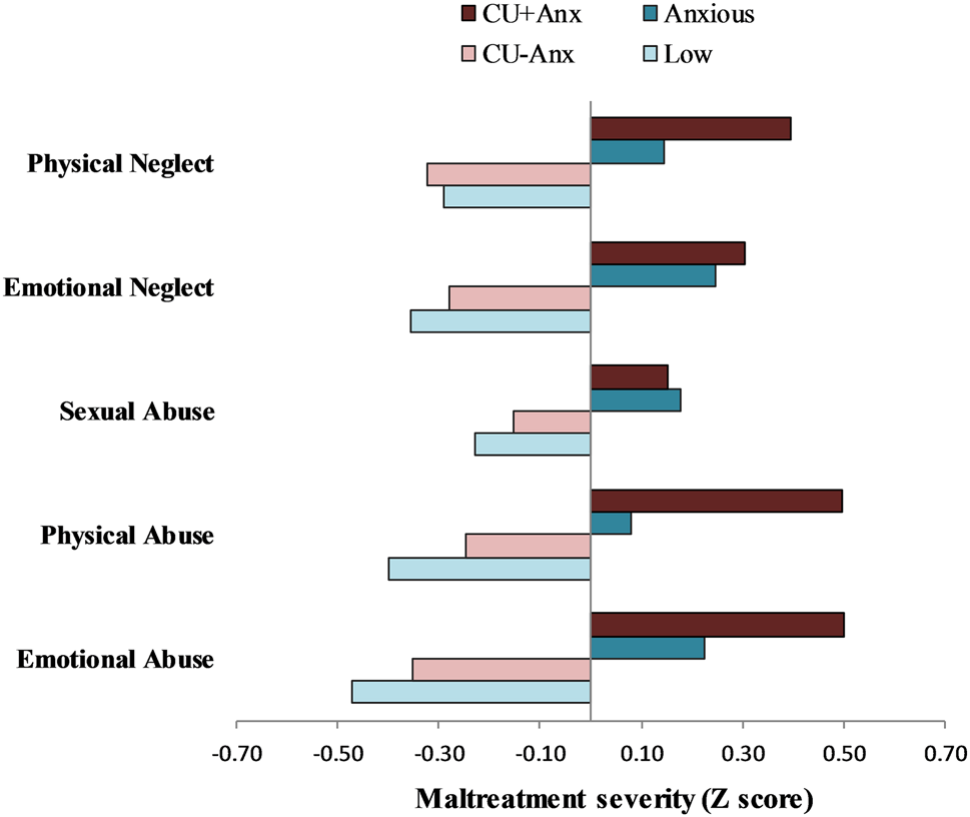
Mean levels of childhood maltreatment severity across groups

Mean levels of maltreatment across groups are shown in Fig. 2. The *CU*+*Anx* group and the *Anxious* group reported comparably high levels of total maltreatment, which differed significantly from the comparably low levels reported by the *CU*−*Anx* and *Low* groups (Table 1). With regard to specific forms of maltreatment, severity was greater in the *CU*+*Anx* group compared to the *CU*−*Anx* group on measures of emotional, physical and sexual abuse as well as physical neglect (*p* < 0.001), with marginal differences for emotional neglect (*p* < 0.05). Across forms of maltreatment, the *CU*+*Anx* group did not differ from the *Anxious* group, while the *CU*−*Anx* group did not differ from the *Low* group.

### Individual functioning

Differences in individual functioning are presented in Table 2. At a mean level, the *CU*+*Anx* group showed the most severe psychiatric symptoms, poorest affective functioning and greatest rates of behavioural risk and disorganised attachment compared to than any other group. All contrasts between the *CU*+*Anx* and *Low* group were significant (*p* < 0.01), except for alcohol use.

**Table 2:** Group comparisons on markers of individual functioning

	Overall sample (*n* = 155)	Low CU		High CU		Omnibus test	Pair-wise contrasts	
		*Low* (*n* = 36)	*Anxious* (*n* = 43)	*CU*−*Anx* (*n* = 36)	*CU*+*Anx* (*n* = 40)		*CU*+*Anx* vs. *CU*−*Anx*	*CU*+*Anx* vs. *Anxious*
							Effect size [95% CI]	Effect size [95% CI]
Psychiatric symptoms								
Self-report								
Depression, *M* (SD)	6.55 (5.00)	3.33 (1.98)	9.00 (4.52)	3.42 (2.93)	9.63 (5.52)	*F *(2,155) = 39.29, *p* < 0.001^a,b^	*** g = 1.37 [0.87, 1.87]	–
Anger, *M* (SD)	7.65 (5.72)	4.44 (3.79)	8.02 (5.19)	5.19 (4.24)	12.33 (5.79)	*F* (2,155) = 28.13, *p* < 0.001^a,b^	*** g = 1.38 [0.88, 1.88]	** g = 0.78 [0.33, 1.22]
PTSD, *M* (SD)	9.77 (6.81)	4.44 (3.78)	11.74 (6.07)	6.31 (4.56)	15.58 (5.99)	*F* (2,155) = 53.61, *p* < 0.001^a,b^	*** g = 1.71 [1.19, 2.24]	^†^ * g* = 0.63 [0.19, 1.07]
Dissociation, *M* (SD)	9.41 (6.13)	6.11 (4.37)	10.53 (5.35)	5.56 (3.62)	14.65 (5.96)	*F *(2,155) = 36.59, *p* < 0.001^a,b^	*** *g* = 1.80 [1.27, 2.34]	** g = 0.72 [0.28, 1.17]
Informant-rated								
GAD, *M* (SD)	4.21 (4.17)	1.52 (1.71)	4.16 (4.62)	4.04 (3.62)	6.82 (4.2)	*F* (2,154) = 18.54, *p* < 0.001^b,c^	** g = 0.70 [0.23, 1.16]	** g = 0.59 [0.15, 1.03]
MDD, *M* (SD)	2.78 (3.74)	0.71 (1.45)	2.65 (3.38)	2.57 (3.00)	5.08 (4.91)	*F* (2,151) = 13.54, *p* < 0.001^b,c^	** g = 0.60 [0.14, 1.06]	** g = 0.58 [0.14, 1.02]
ODD, *M* (SD)	2.91 (4.21)	0.56 (1.48)	1.46 (2.47)	4.06 (4.01)	5.63 (5.59)	*F* (2,152) = 10.10, *p* < 0.001^a,b,c^	–	*** *g* = 0.97 [0.51, 1.42]
CD, *M* (SD)	1.38 (2.79)	0.11 (0.40)	0.56 (1.10)	1.83 (2.64)	3.08 (4.28)	*F *(2,151) = 8.18, *p* < 0.001^b,c^	–	*** g = 0.81 [0.37, 1.26]
ASPD, *M* (SD)	2.09 (3.50)	0.26 (0.82)	0.93 (1.72)	2.77 (3.89)	4.52 (4.55)	*F *(2,151) = 14.54, *p* < 0.001^b,c^	–	*** g = 1.05 [0.59, 1.51]
ADHD, *M* (SD)	7.85 (9.19)	2.14 (4.40)	5.14 (8.96)	9.40 (7.47)	14.73 (9.57)	*F* (2,152) = 21.41, *p* < 0.001^b,c^	** g = 0.61 [0.57, 1.48]	*** g = 1.03 [0.57, 1.48]
Behavioural risk markers								
Alcohol use, *M* (SD)	4.99 (5.31)	5.14 (4.65)	4.71 (4.88)	4.34 (4.20)	5.78 (7.15)	*X* ^2^(3,150) = 1.38, ns	–	–
Drug use, *M* (SD)	3.49 (6.91)	1.89 (4.86)	2.69 (5.68)	3.48 (6.42)	5.97 (9.47)	*X* ^2^(3,150) = 23.08, *p* < 0.001^b^	*** OR = 2.17 [1.27, 3.71]	** OR = 2.18 [1.32, 3.60]
Suicidal ideation (%)	15.1	0	12.0	14.3	33.3	*X* ^2^ (3,152) = 16.84, *p* < 0.001^b^	–	^†^ OR = 3.70 [1.17, 11.65]
Suicide attempt (%)	10.3	0	7.0	11.0	22.5	*X* ^2^ (3,152) = 11.10, *p* < 0.01^b^	–	–
Unsafe sex (%)	37.1	22.7	27.6	34.5	64.0	*X* ^2^(3,105) = 10.32^†^	^†^ OR = 3.38 [1.10, 10.35]	** OR = 4.67 [1.47, 14.79]
Affective functioning								
Irritability, *M* (SD)	4.09 (3.71)	2.51 (2.67)	4.48 (4.10)	2.82 (2.54)	6.21 (3.93)	*F* (2,150) = 12.77, *p* < 0.001^b^	*** g = 0.99 [0.52, 1.47]	–
Alexithymia *M* (SD)	14.63 (6.38)	10.39 (3.42)	16.55 (6.68)	12.88 (5.13)	18.05 (6.59)	*F* (2,151) = 21.49, *p* < 0.001^b^	*** *g* = 0.85 [0.38, 1.323]	–

N.B. Analyses control for sex. Hedge’s *g* guidelines for effect size: *g* of 0.20 = small, 0.50 = medium, 0.80 = large

*GAD* generalised anxiety disorder, *MDD* major depressive disorder, *ODD* oppositional defiant disorder, *CD* conduct disorder, *ASPD* antisocial personality disorder, *ADHD* attention-deficit hyperactivity disorder, *OR* odds ratio

^†^ *p* < 0.05, ** *p* < 0.01, *** *p* < 0.001

^a^ *CU*−*Anx* vs *Anxious* contrast significant at least at *p* < 0.01; ^b^ *CU*+*Anx* vs *Low* contrast significant at least at *p* < .01; ^c^ *CU*−*Anx* vs *Low* significant at least at *p* < 0.01

#### Psychiatric symptoms

**Fig. 3:**
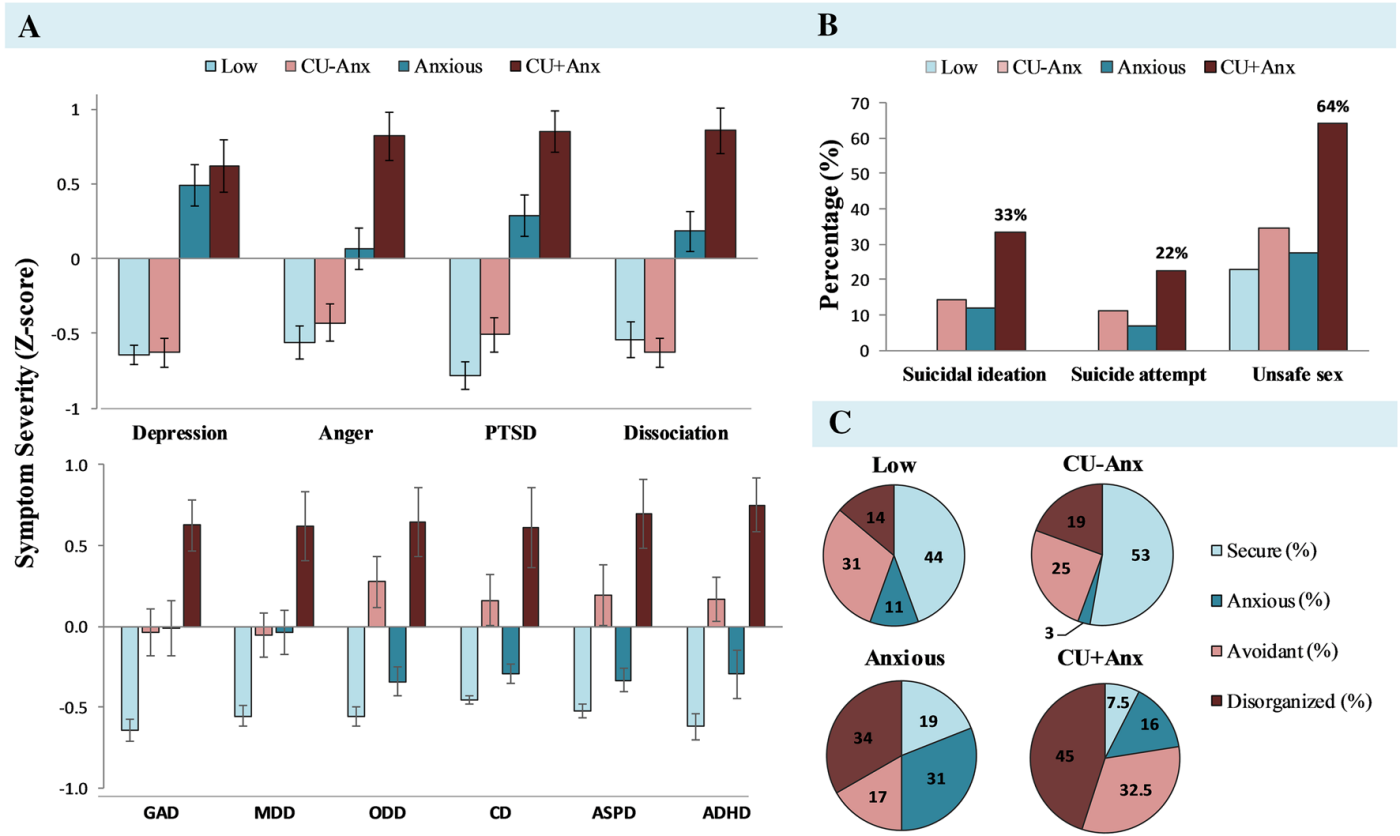
Group differences on levels of psychiatric symptomatology, behavioural risk and attachment style. **a** Standardised mean levels of self-report (TSCC-A; top-half) psychological distress and informant-report (ASI; bottom-half) psychiatric symptomatology across groups. **b** Percentage of endorsement of behavioural risk items across groups. **c** Attachment style classification across groups. *GAD* generalised anxiety disorder, *MDD* major depressive disorder, *ODD* oppositional defiant disorder, *CD* conduct disorder, *ASPD* antisocial personality disorder, *ADHD* attention-deficit hyperactivity disorder

The *CU*+*Anx* group reported significantly higher internalising symptoms compared to the *CU*−*Anx* group (see Fig. 3a), based on both self-reported (i.e. depression) and informant-rated outcomes (i.e. GAD and MDD). As predicted, the two variants did not differ from one another in externalising behaviours—showing comparable symptoms of conduct disorder, oppositional defiant disorder, and antisocial personality disorder. Both CU groups scored significantly higher on these externalising problems compared to either the *Anxious* or *Low* comparison groups. Interestingly, *CU*+*Anx* youth differed significantly from all other groups in levels of self-reported psychological distress (i.e. anger, post-traumatic stress and dissociation) as well as informant-rated ADHD symptomatology—with differences being moderate to large across these domains. The *CU*−*Anx* and *Low* groups showed comparably (low) levels of psychological distress.

#### Behavioural risk markers

There was no significant main effect of group on alcohol use. The *CU*+*Anx* group reported higher drug use than the *CU*−*Anx* group (*p* < 0.001, OR = 2.17) and *Anxious* group (*p* < 0.01, OR = 2.18). Endorsement of behavioural risk items across groups related to suicidality and unsafe sex are graphically presented in Fig. 3b. Significant main effects were found for suicidal ideation, suicide attempt and unsafe sex. In the *CU*+*Anx* group, 33.3% of participants reported having thought of committing suicide in the past year and 22.5% attempted suicide, compared to 14.3% ideation and 11% attempt in the *CU*−*Anx* group. Rates of suicidal ideation and attempt within the *CU*+*Anx* group were also considerably higher than within the *Anxious* and *Low* groups. In addition, of those who had sexual intercourse, more than half (64%) in the *CU*+*Anx* group reported not using a condom or other contraceptive during their last sexual encounter, compared to 34.5% in the *CU*−*Anx* group, 27.6% in the *Anxious* group and 22.7% in the *Low* group.

#### Attachment style

Attachment style differed significantly across groups, *X* ^2^(9,154) = 38.10, *p* < 0.001. As can be seen in Fig. 3c, the most striking difference relates to the proportions of secure vs disorganised attachment across groups. The *CU*+*Anx* group were predominantly characterised by disorganised (45%) and avoidant attachment (32%) styles, with only 7.5% showing secure attachment, the lowest proportion relative to any other group. The *Anxious* group were predominantly characterised by disorganised (34%) and anxious attachment (31%) styles, with 19% showing secure attachment. In contrast 53% and 44% of the *CU*−*Anx* and *Low* groups, respectively, were classified as securely attached.

#### Affective functioning

The two variants of CU youth differed significantly on both measures of affective functioning, with the *CU*+*Anx* group showing higher levels of irritability (*p* < 0.001, *g* = 0.99) and alexithymia (*p* < 0.001, *g* = 0.85). In contrast, the *CU*+*Anx* group did not differ from the *Anxious* group on either measure of affective functioning. The *CU*−*Anx* group showed a profile of affective functioning similar to that of the *Low* group.

### Post hoc power analysis

The sample size in our study is consistent with the extant literature on variants of CU youth in high-risk samples (e.g. [15, 19, 22]), whereby elevated rates of developmental adversity and psychiatric symptomatology result in increased power to detect effects (i.e. as opposed to general population samples). Nevertheless, we performed a post hoc analysis to ensure that we were appropriately powered for the analyses undertaken. Based on post hoc G*Power calculations, with a sample size of *n* = 155, four groups and moderate-to-large effect sizes for all outcome variables, we found that achieved power exceeded 0.85 across analyses.

## Discussion

This study systematically characterised variants of CU youth in a high-risk community sample. Specifically, we compared youth who presented with similarly high levels of CU traits, but different levels of co-occurring anxiety (i.e. *CU*+*Anx* vs *CU*−*Anx*) on maltreatment history, psychiatric symptomatology and broad markers of individual functioning. The use of multiple informants was a key strength of our study, with multi-rated assessments used in both construction of CU groups as well as the examination of individual functioning domains. We highlight here three main findings. First, youth with *CU*+*Anx* were characterised by more severe histories of childhood abuse and neglect compared to *CU*−*Anx* youth. Second, while variants of CU youth did not differ on levels of externalising problems (e.g. oppositional defiant and conduct disorder symptoms), the *CU*+*Anx* group presented with significantly elevated levels of psychological distress (i.e. depression, anger, dissociation and PTSD symptoms), insecure attachment, affective dysregulation and behavioural risk. Third, the inclusion of an *Anxious* comparison group revealed widespread similarities in trauma history and individual functioning between CU+*Anx* youth and those low in CU but high in anxiety. Generally, *CU*+*Anx* youth seemed to experience a ‘double hit’ of negative outcomes associated with CU on the one hand, and anxiety on the other. They also showed additional vulnerabilities compared to youth who presented with either *CU* or *Anxiety* alone, including more severe feelings of anger and dissociation, elevated ADHD symptoms, greater drug use, engagement in unsafe sex and higher suicide risk. Overall, the identification of distinct patterns of co-occurring psychiatric, emotional and behavioural markers associated with variants of CU youth have important and immediate clinical applications for informing risk assessment and treatment formulation.

### Childhood maltreatment robustly discriminates between variants of CU youth

As hypothesised, childhood maltreatment emerged as a key factor discriminating variants of CU youth, with *CU*+*Anx* youth reporting more severe trauma histories compared to the *CU*−*Anx* group across all individual forms of abuse and neglect. This finding is consistent with prior research that examined maltreatment as a global construct (or as part of a wider adversity measure; e.g. [6, 14, 15]). While previous studies that have compared variants of CU youth on individual forms of maltreatment (e.g. [16–18]) have shown some inconsistencies regarding which precise forms of maltreatment reliably differentiate *CU*+*Anx* and *CU*−*Anx* groups, all have reported more pervasive maltreatment experiences in the *CU*+*Anx* group—which is broadly in line with our findings. In contrast to previous studies, we additionally compared maltreatment profiles against two comparison groups (i.e. *Low* and *Anxious*). While *CU*+*Anx* youth reported comparable levels of abuse and neglect to youth presenting with high anxiety but low CU (i.e. the *Anxious* group) the *CU*−*Anx* group did not differ in maltreatment history from those showing low CU and low anxiety (i.e. *Low* group).

### CU+*Anx* indexes a particularly vulnerable group of individuals

Youth with *CU*+*Anx* presented with the highest mean levels of psychological distress across all domains examined, in line with adult data on individuals who score high on psychopathy and anxiety [8] as well as youth data on CU groups [6, 7, 14, 15]. Additionally, the *CU*+*Anx* group was characterised by significantly elevated behavioural risk, including increased drug use, feelings of suicidality and engagement in unsafe sex. Alarmingly, one third of youth in the *CU*+*Anx* group in this high-risk sample reported having seriously considered committing suicide in the past year, and almost one fourth reported attempting suicide. Rates of unsafe sex were also high in the *CU*+*Anx* group, with more than half of youth reporting not using a condom or other contraceptive during their last sexual intercourse. These figures are disturbing given the known associations between unsafe sexual behaviours and adverse health outcomes [48], and suggest the *CU*+*Anx* group is highly vulnerable across multiple domains.

Our exploratory measures delineated additional differences across variants of CU youth in areas of affective functioning and attachment to close others. Elevated levels of irritability and anger in the *CU*+*Anx* group are consistent with the notion that this variant features increased emotional expression and reactivity [6, 17]. Furthermore, attachment disorganisation, an established sequel of childhood maltreatment [49], was found to be most common in youth with *CU*+*Anx*, while *CU*−*Anx* featured predominantly a secure attachment style. To our knowledge, this is the first study to have examined current patterns of attachment styles across CU groups. Finally, increased levels of alexithymia observed in *CU*+*Anx* (and *Anxious* youth) compared to *CU*−*Anx* youth may also reflect the developmental impact of childhood maltreatment on emotional arousal and functioning. The finding related to alexithymia warrants further investigation, as it may offer clues as to why individuals with *CU*+*Anx* share behavioural features with those with *CU*−*Anx* (in other words, the present with a ‘behavioural phenocopy’), yet appear emotionally reactive in a way that *CU*−*Anx* are not. High levels of alexithymia are associated with an inability to describe and identify emotions, rather than an inability to *experience* emotional arousal. This means that although these individuals may experience heightened affect in response to another person’s distress, their ability to display socially appropriate responses may be compromised, leading them to appear callous and uncaring. The finding that *CU*+*Anx* reported the highest levels of dissociative symptoms compared to any other group may lend additional support for this hypothesis, as do prior reports of lack of emotional ‘clarity’ within this group [28]. In contrast, adults with primary psychopathy and youth with CU−*Anx* have been shown to be typically characterised by low emotional arousal to other people’s distress [17].

### *CU*+*Anx* youth share many similarities with *Anxious* youth

The inclusion of two comparison groups enabled us to compare variants of CU youth to low CU youth who also vary in their levels of anxiety. Interestingly, we found that *Anxious* youth, albeit lower in levels of externalising problems, reported similar levels of childhood trauma, emotional difficulties and psychological distress to *CU*+*Anx* youth. Consequently, an important question that emerged from the present data related to why some youth with a history of trauma presented with both high levels of CU and anxiety (i.e. *CU*+*Anx*) while others only present with high anxiety (i.e. *Anxious* group). One possibility is that youth with *CU*+*Anx* have additional genetic vulnerability to externalising disorders/impulsivity, as is suggested by their substance use, suicidal ideation and sexual behaviour profile. It is also possible that *CU*+*Anx* youth may be exposed to additional environmental risk factors relative to *Anxious* youth, that were not captured in the current study (e.g. bullying-victimisation). Longitudinal investigations charting children who have experienced maltreatment, but who come from families characterised by different levels of externalising problems, could shed light into this issue.

### Research and clinical implications

The present findings highlight the need to differentiate between variants of CU youth. Supplementing measures of CU traits with an assessment of anxiety can offer important information for both clinicians and researchers. Failure to assess levels of anxiety among youth with high CU traits may obscure the diverse constellations of needs and risk factors associated with subgroups of individuals presenting with elevated CU traits. Equally, the current findings highlight that experiences of childhood maltreatment markedly differ between variants of CU youth. In research and clinical settings, developmental adversity is not always assessed concurrently with CU traits in youth [14]. An increased awareness of maltreatment as a possible risk factor for *CU*+*Anx* may be helpful in informing risk assessment and suitable intervention strategies. Importantly, the findings indicate that focussing on conduct problems or antisocial behaviour alone is unlikely to discriminate between variants of CU youth, as they tend to present similarly on these domains.

Youth with *CU*+*Anx* represent a high-risk clinical group characterised by more severe developmental trauma, concurrent psychiatric symptomatology, affective dysfunction, risk behaviours and suicide risk. For these youths, therapeutic approaches that include the experience of trauma in the treatment formulation, such as trauma-focussed CBT and similar evidence-based interventions, may be warranted. Equally, interventions addressing conduct problems in youth with *CU*+*Anx* may need embedding in a wider therapeutic intervention addressing other internalising problems, particularly anxiety and depression. High rates of disorganised attachment in this group are likely to predict poor interpersonal functioning, and will be relevant to the clinician challenged with establishing appropriate boundaries alongside an effective therapeutic alliance. Finally, risk assessments will need to pay particular attention to engagement in risky behaviours (e.g. drug use) and increased risk of suicidality as these were strongly associated with *CU*+*Anx*. More broadly, our findings support a growing emphasis in the field on CU traits as a cross-disorder construct [50, 51], which needs to be more fully considered within the broader context of different forms of psychopathology and risk behaviours across both research and clinical settings.

### Limitations

The findings of present study should be interpreted in light of several limitations. First, CU traits are a dimensional construct, not a taxon. As we wished to compare variants of CU traits, a categorical approach provided an effective means of communication and this way of characterising children is also directly relevant for informing clinical practice. In future, studies may benefit from using dimensional information to supplement categorical approaches. Furthermore, although the measure used in our study to index CU traits (i.e. the ICU) has been commonly employed in the literature on variants of CU youth as well as being shown to possess good factor structure, construct and predictive validity in a range of populations [52–56], some concerns have been raised about aspects of its psychometric properties [57] so that results will need to be replicated using an independent measure of CU traits. Second, the anxiety measure used in this study to define groups was taken from the same questionnaire as our self-reported outcomes of psychological distress, which raises issues of shared-method variance. However, it is important to note that variants of CU youth were also found to differ on levels of internalising problems (i.e. symptoms of generalised anxiety and major depressive disorder) based on ratings from independent informants (i.e. teachers/key workers). Third, while inclusion of a measure of childhood maltreatment provided a temporal proxy for the effect of developmental adversity on *CU*+*Anx*, the cross-sectional nature of the study meant that we were unable to establish the causality of effects found. However, the consistency with which childhood maltreatment has been found to differentiate between variants of CU youth across our study and that of the extant literature (e.g. [14–18]) considerably adds confidence to this finding. Despite this, it is important to note that while the data seem to suggest that *CU*+*Anx* may be more environmentally driven than *CU*−*Anx*, it was not possible to remove potential genetic confounds from our design (e.g. youth high in CU may be more likely to have parents high in psychopathic traits, who are also more likely to maltreat them). Genetically informative designs may be particularly effective in examining the contribution of such influences (e.g. [58]). Fourth, while post hoc analyses confirmed that we were appropriately powered for all analyses undertaken, sample size limitations meant that we were only able to enter sex as a free-standing covariate. In future, the use of larger samples will make it possible to examine whether sex moderates associations between variants of CU youth and markers of individual functioning. Finally, even though sampled from the community, youth in our study came predominantly from high-risk, multi-problem families. As a result, further research is needed to establish the extent to which findings may generalise to the wider population.

### Future directions

The present findings point to a number of directions for future research. First, longitudinal, prospective research is needed to gain a more mechanistic understanding of processes underlying variants of CU traits in youth. Longitudinal studies may also help determine whether variants are predictive of different developmental trajectories and outcomes over time, particularly in relation to frequency and nature of violence, suicidality, and mental health problems. Indeed, efforts to map variants longitudinally are already beginning to emerge [7, 23, 26, 27]. Second, examining the timing of maltreatment experiences may be important for understanding how *CU*+*Anx* develops and identifying whether developmental windows exist where the effect of maltreatment is more pronounced. Third, *CU*+*Anx* may represent a ‘phenocopy’ of *CU*−*Anx*, but the origins of CU and the underlying neurocognitive mechanisms for the two variants may differ. A number of studies have provided support for differences in behavioural performance across variants on measures of emotional processing and behavioural activation [6, 16, 17, 28]. Future neurocognitive studies would benefit from direct comparisons of *CU*+*Anx* with anxious individuals, as well as use of tasks that investigate processes that should be compromised in CU, but not in anxiety. Fourth, given that CU traits are known to be moderately associated with conduct problems (e.g. *r* = 0.54 in our study) it will be important in future to establish to what extent these co-occurring symptoms may be driving observed differences between *CU*+*Anx* vs *Anx* only youth. From a research perspective, it is notable that we found group differences in the ratio of males to females across variants of CU youth in this community sample. While the group of youth with *CU*−*Anx* contained disproportionately more boys, the *CU*+*Anx* group had a more balanced male to female ratio (slightly greater number of girls). Moreover, the *Anxious* group featured predominantly females. These findings are in line with previous work examining variants of CU youth [31]. Interestingly, another study has reported that psychopathic personality traits are associated with a history of trauma in young female offenders [59]. Future studies should test whether the difference in sex ratio is a reliable finding and whether the experience of trauma may represent a particularly potent risk factor for CU+*Anx* in girls. Finally, research is needed to inform the development of more tailored interventions as well as to evaluate whether the application of differing strategies may be more effective than a ‘one size fits all’ intervention. This is especially important given the dearth of programmes specifically validated on youth with CU traits [17]. Together, studies addressing these future directions will contribute to a greater understanding of the nature and significance of variants of CU youth.
